# Exploring the Effects of Organic Matter Characteristics on Fe(II) Oxidation Kinetics in Coastal Seawater

**DOI:** 10.1021/acs.est.1c04512

**Published:** 2022-01-25

**Authors:** J. Magdalena Santana-Casiano, David González-Santana, Quentin Devresse, Helmke Hepach, Carolina Santana-González, Birgit Quack, Anja Engel, Melchor González-Dávila

**Affiliations:** † Instituto de Oceanografía y Cambio Global, Universidad de Las Palmas de Gran Canaria, Campus de Tafira, 35017 Las Palmas, Spain; ‡ Université de Brest, CNRS, IRD, Ifremer, LEMAR, F-29280 Plouzane, France; § GEOMAR—Helmholtz Centre for Ocean Research Kiel, Düsternbrooker Weg 20, 24105 Kiel, Germany

**Keywords:** iron(II), oxidation kinetics, Macaronesia, coastal seawater, CDOM, FDOM

## Abstract

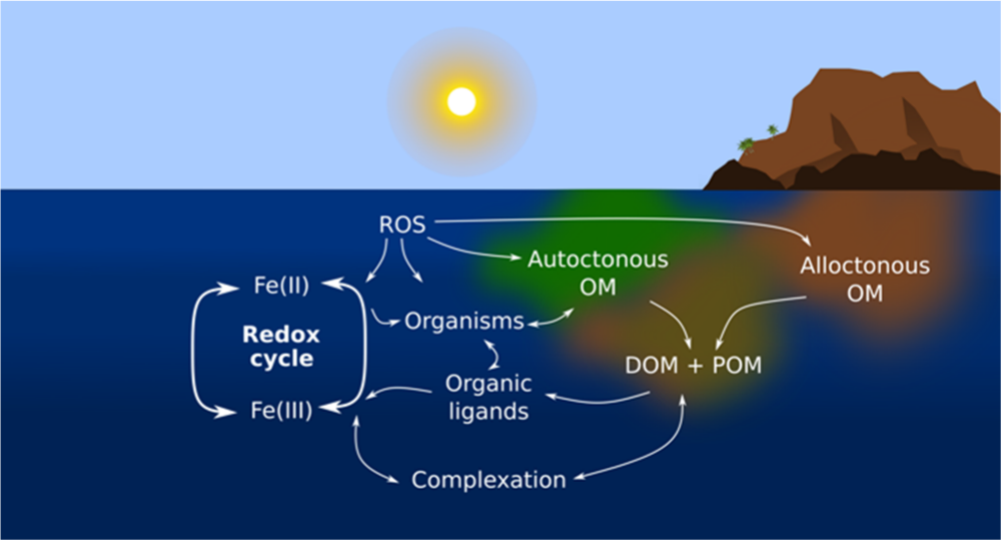

The iron(II) oxidation kinetic process was studied at 25 stations in coastal seawater of the Macaronesia region (9 around Cape Verde, 11 around the Canary Islands, and 5 around Madeira). In a physicochemical context, experiments were carried out to study the pseudo-first-order oxidation rate constant (*k*′, min^–1^) over a range of pH (7.8, 7.9, 8.0, and 8.1) and temperature (10, 15, 20, and 25 °C). Deviations from the calculated *k*_cal_^′^ at the same T, pH, and S were observed for most of the stations. The measured *t*_1/2_ (ln 2/*k*′, min) values at the 25 stations ranged from 1.82 to 3.47 min (mean 1.93 ± 0.76 min) and for all but two stations were lower than the calculated *t*_1/2_ of 3.21 ± 0.2 min. In a biogeochemical context, nutrients and variables associated with the organic matter spectral properties (CDOM and FDOM) were analyzed to explain the observed deviations. The application of a multilinear regression model indicated that *k*′ can be described (*R* = 0.921 and SEE = 0.064 for pH = 8 and *T* = 25 °C) from a linear combination of three organic variables, *k*′^OM^ = *k*_cal_^′^ −0.11* TDN + 29.9*b_DOM_ + 33.4*C1_humic_, where TDN is the total dissolved nitrogen, *b*_DOM_ is the spectral peak obtained from colored dissolved organic matter (DOM) analysis when protein-like or tyrosine-like components are present, and C1_humic_ is the component associated with humic-like compounds obtained from the parallel factor analysis of the fluorescent DOM. Results show that compounds with N in their structures mainly explain the observed *k*′ increase for most of the samples, although other components could also play a relevant role. Experimentally, *k*′ provides the net result between the compounds that accelerate the process and those that slow it down.

## Abstract 2

Fe(II) persistence in the marine environment is conditioned by the characteristics of the dissolved organic matter.

## 1 Introduction

Iron (Fe) is a trace element whose speciation and concentration in the marine environment are affected by multiple abiotic and biotic processes.^1^ Redox and complexation reactions control its solubility and therefore the fraction of dissolved and bioavailable Fe.^2^ Processes such as photo-oxidation are a key factor in the control of Fe speciation in surface waters, particularly in areas subject to high irradiation and organic matter content.^3^ Iron is essential for organisms and plays an important role in the functioning of marine ecosystems.^1^ Marine organisms have developed different strategies to assimilate Fe, ranging from the reduction of metal on the cell surface to the release of ligands that complex Fe.^4^ These ligands become part of the organic matter pool. The amount and type of organic matter present in the medium control the speciation of Fe.^5^ Dissolved organic matter (DOM) affects the Fe organic complexation, with the colloidal and particulate organic matter (COM and POM) acting as net dissolved Fe sinks.^6,7^

In the ocean, the thermodynamically stable form of Fe is the oxidized ferric iron (Fe(III)), with the soluble fraction in its majority complexed with organic ligands.^8^ However, ferrous iron (Fe(II)) can also be found in these conditions.^9^ In surface waters, many authors attribute it to photo-reduction processes in which complexed Fe(III), Fe(III)L, is involved.^10^ Abiotic redox reactions can also occur in the absence of light, causing the reduction of Fe(III) induced by oxidation of organic matter.^11^ In anoxic areas and hydrothermal vents, Fe(II) is the predominant form and as soon as these waters are mixed with oxygen-rich waters, Fe(II) tends to oxidize.^12,13^ The persistence of Fe(II) coming from hydrothermal vents or anoxic sediments is attributed to the complexation with organic ligands and the formation of iron sulfide (FeS).^12,14^ In the water column, in situ processes such as the remineralization of POM during denitrification are possible sources of Fe(II).^15^ Particle-bound Fe(III) can be reduced to Fe(II) by reduced sulfur compounds produced within reducing microenvironments of particles.^16^ In the continental margin, the effect of allochthonous matter is important in the Fe(II) behavior controlling its oxidation.^17,18^

Knowing how long Fe(II) can remain in solution is one of the challenges in marine chemistry. This knowledge is required to understand the chemical behavior of this trace metal in the marine environment especially when considering that Fe(II) partakes and has played an essential role in the development of marine organisms since primordial times.^19^ Information on the rate of oxidation of Fe(II) and the half-life time in the ocean is therefore essential for global biogeochemical models.^20^

This work aimed to study the Fe(II) oxidation kinetic constants in coastal seawater and to investigate the possible factors that could contribute to explain the different persistence of Fe(II) in the marine environment. These studies provide a rate constant (log *k*′) and a half-life time (*t*_1/2_) data set under different pH and temperature conditions, which can be incorporated into ocean biogeochemical Fe models. The *k*′ and *t*_1/2_ values at a fixed pH and *T* were also determined for every sample. This allows for the identification of non pH and *T* effects on the oxidation rate constants. Moreover, spectral parameters and ratios obtained from the colored and fluorescent DOM (CDOM and FDOM) were used to provide an equation that accounts for the deviation between measured *k*′ and calculated *k*_cal_^′^. While the focus of this study is the identification of which variables associated with the organic matter spectral properties (CDOM and FDOM) can explain the deviations of the measured *k*′ from the calculated *k*_cal_^′^ at the same *T* and pH, additional attention should be given in future studies to the effects of other parameters such as Fe(II) and organic ligand concentration ratios and the effect of photogenerated compounds.

## 2 Materials and Methods

### 2.1 Sampling Protocol

**Figure 1:**
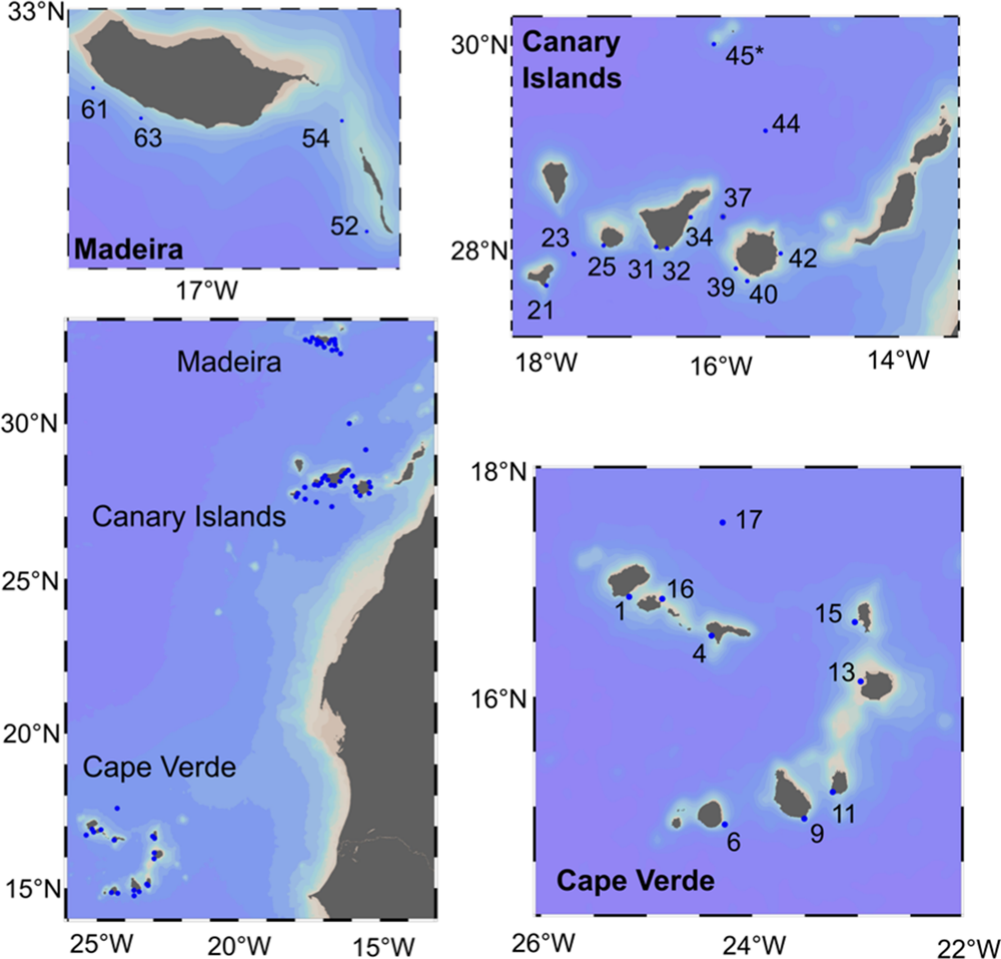
Map of the archipelagos. Cape Verde Island, Canary Islands, and Madeira region. Station 45* (Selvages) belongs to Madeira.

Samples were collected onboard the RV POSEIDON during the POS533 cruise. The cruise started on the 28th of February 2019 in Mindelo, Sao Vicente (Cape Verde), and ended on the island of Gran Canaria (Canary Islands) on the 22nd of March 2019. The cruise was divided into three sets of stations (Figure 1). The first set was located in the Cape Verde archipelago, the second set was positioned around the Canary archipelago, and the third set encompassed the Madeira area. The number and sampling locations are indicated in Table 1.

**Table 1:**
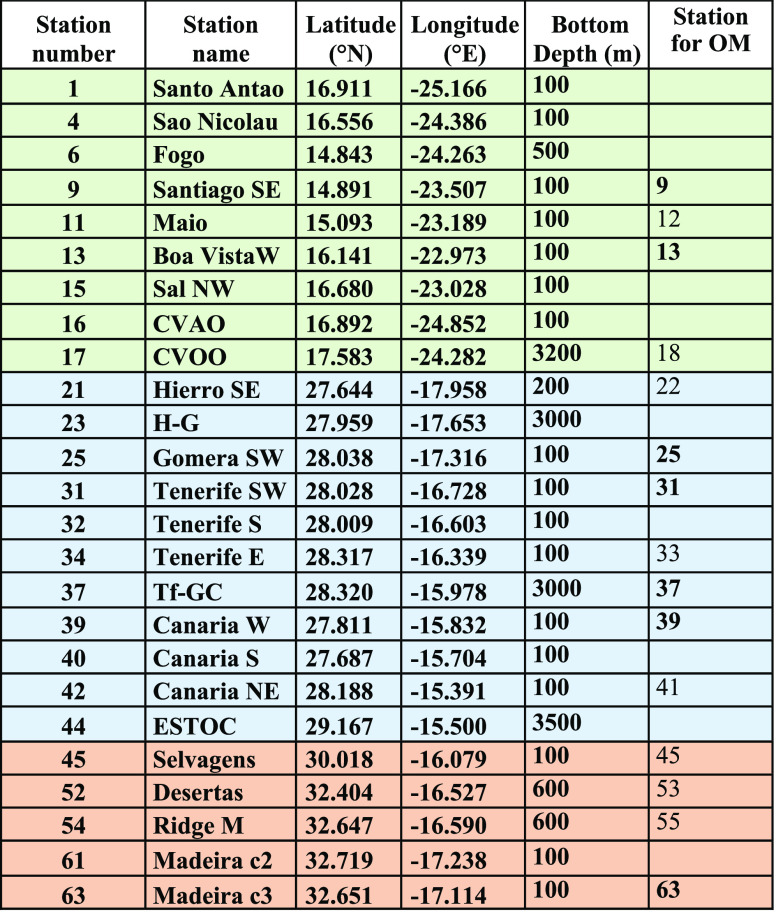
Station Number, Name, Location, and Bottom Depth of the sites where Fe(II) oxidation kinetics samples were collected during the POS533 Cruise^a^

a Stations for OM are the closest stations where organic matter variables were collected.

At each station, trace metal samples were collected at 20 m depth using a Teflon pump (Furon) with a 40 m Teflon tube connected with an AcroPak 1500 capsule w/Supor Memb 0.8/0.2 μm filter. The pump was left for 15 min in flowing seawater to rinse the inner hose, and the 1 L sample was collected in LDPE bottles (Nalgene) and stored at −20 °C until the land-based laboratory analysis. The material was previously cleaned following the trace metal GEOTRACES protocol,^21^ and the experiments were performed in ISO Class 5 laminar flow hoods within an ISO Class 6 clean lab (QUIMA-IOCAG TM lab).

A lowered SeaBird SBE 9-plus CTD system equipped with two sets of pumped sensors measuring conductivity, temperature, and oxygen at 24 Hz was used. The CTD was mounted on a SeaBird rosette with 12 bottles (10 L) which were used to take discrete water samples for sensor calibration and biogeochemical parameters.^22^ The biogeochemical parameters sampled were nitrate + nitrite (NO_3_ + NO_2_), phosphate (PO_4_), silicates, dissolved and particulate organic carbon (DOC, POC), total dissolved and particulate nitrogen (TDN, PN), CDOM, and FDOM. The mixed layer depth was deeper than 20 m.^22^ Information about sampling and analysis is included in the Supporting Information.

### 2.2 UV Irradiation

An Ace Glass-incorporated ultra-violet photo-oxidation unit which operates at 120 V, 60 Hz was used to UV irradiate the samples. The quartz UV lamp works at 430 mA, 254 nm, and 3.5 W. For the irradiation procedure, each sample was placed in a 100 mL quartz tube and 12 tubes were irradiated at the same time. The samples were irradiated for 4 h. They were then kept in the dark and let to equilibrate with atmospheric air in a laminar flow for more than 72 h before the analysis.

### 2.3 Oxidation Kinetic Experiments

The oxidation kinetic experiments were carried out in a 60 mL LDPE container within a double-wall glass thermo-regulated cell, connected to a thermostatic bath (Julabo), as described in previous studies.^23,24^ For each oxidation kinetic study, 25 mL of the seawater sample was used. The initial concentration of added Fe(II) was 0.97 nM. Previous studies have shown that the inorganic Fe(II) oxidation rate constant is independent of the added Fe(II) concentration.^25,26^ The experiments were carried out at a fixed free-scale pH_F_ = 8 with temperatures of 10, 15, 20, and 25 °C to get the temperature dependence and similarly, at a fixed temperature of 25 °C with pH_F_ of 7.8, 7.9, 8.0, and 8.1 to get the pH dependence. In each oxidation kinetic experiment, the pH was controlled using a Titrino 719 (Metrohm) which automatically added 0.01 M hydrochloric acid (HCl, Panreac Hiperpur-plus). The pH electrodes were calibrated using a Tris buffer solution.^27^ All the experiments were carried out under O_2_ saturated conditions to avoid oxygen limitation. Experiments were performed in the dark to avoid the generation of reactive oxygen species (ROS) from photo-oxidation processes and the photo-transformation of Fe.

The concentration of total dissolved Fe(II), TdFe(II), in the samples for the kinetics experiments, was determined using the flow injection analysis by chemiluminescence (FIA-CL) technique with a FeLume system (Waterville Analytical)^28^ as described in previous studies.^12,23^ The FIA-CL technique uses luminol as the reagent. 5 L of the luminol reagent was prepared using 2.71 × 10^–4^ M of 5-amino-2,3-dihydro-1,4-phthalazinedione (Sigma), 4.93 × 10^–2^ M of Na_2_CO_3_ (Sigma-Aldrich), and 0.4 M of previously distilled 25% NH_3_ (Panreac). The final pH was adjusted to 10.4 by adding 0.06 M Q-HCl. The luminol solution was stored in the dark due to its light sensitivity.

The kinetic studies were carried out in a thermo-regulated cell connected to a thermostatic bath (Julabo) with control to ±0.01 °C. For each study, the seawater sample was acclimated to the desired temperature (in situ and 25 °C). When the temperature was stable, the pH_F_ for the sample was measured.^27^ For each oxidation kinetic analysis, 50 mL of seawater was used. The seawater was placed in the thermostated cell, and the magnetic stirrer was switched on for 1 h to attain oxygen concentration equilibrium. When the solution stabilized at the desired temperature and pH, the sample line was introduced into the reaction cell. The samples were automatically mixed with the buffer just before being introduced into the detector. The sample plus luminol (1 mL min^–1^ flux) and the ammonium acetate buffer (0.04 M ammonium acetate adjusted to pH 5.5 with acetic acid at 0.125 mL min^–1^ flux) were introduced into the detector with a peristaltic pump. This modification provided continuous registration of the measure. This was followed by the Fe(II) addition, and the time was registered.

Iron(II) oxidation kinetic studies in different aqueous media and conditions^25,28−37^ have shown that the rate of oxidation with O_2_ can be expressed as an apparent rate constant (*k*_app_), regardless of the mechanism that describes the process (eq 1).
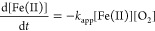
1where *k*_app_ = *k*[OH^–^]^2^. The brackets indicate the total molar concentration.

The inorganic (i) and organic complexation (L) of Fe influences the kinetics rate constant,^29,34^ and the apparent rate constant includes the contribution of the inorganic and organic species of Fe(II)
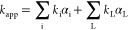
2

When the reaction is studied in excess O_2_, the reaction can be considered pseudo-first-order (eq 3).
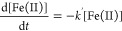
3where *k*′ = *k*_app_[O_2_] in s^–1^ and *k*_app_ is expressed in M^–1^ s^–1^.

The slope corresponding to the plot of Ln [Fe(II)] versus time determines the *k*′ (min^–1^). In the Supporting Information (Figure S1), the lineal relationship is shown for St1.^12,23,28^

The calculated *k*_cal_^′^ value was obtained from an empirical equation^35^ for known conditions of temperature, pH, and salinity (eq 4). This equation is valid from 0.5^26^ to 200 nM.^35^ In this study, the experimental conditions were pH = 8, *T* = 25 °C, and *S* = 35.
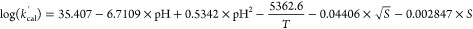
4

The extended equation^12^ calculated for a higher range of low temperatures applicable to deep waters (until 2 °C) can also be used.

### 2.4 CDOM and FDOM Study

For the CDOM study, the *a*_254_ and *a*_325_ absorption coefficients at the wavelengths 254 and 325 nm, respectively,^38^ were selected together with the slope ratio (*S*_R_) and the E2/E3 ratios.^38,39^ The dimensionless *S*_R_ was calculated from the ratio of the slope of the shorter wavelength region (275–295 nm) to that of the longer wavelength region (350–400 nm). The E2/E3 was calculated as the ratio of absorption at 250 to 365 nm.^39−41^ From the FDOM study, the fluorescence peaks,^42^ the humification index (HIX), and the biological index (BIX) were determined^43,44^ and the parallel factor analysis (PARAFAC) was applied. The variable *b*_DOM_ was obtained from the fluorescence peak analysis. *b*_DOM_ is a peak that represents tyrosine-like and protein-like components (with fluorescence Ex/Em = 275/305), and it has been interpreted as a material of autochthonous origin.^45^

The corrected excitation emission matrices (EEMs), the HIX^43^ and BIX,^44^ were used to determine the relative degree of humification and autotrophic productivity of fluorescent CDOM, respectively. HIX is the ratio of the fluorescence over Em 434–480 nm to that over 300–346 nm (at Ex 255 nm). BIX is the ratio of the fluorescence at Em 380 nm to that at 430 nm (at Ex 310 nm). HIX is the estimate of the degree of DOM maturation with an increase in red-shifted emission which presumably arises from increasing conjugation (and possibly aromatization) of DOM.^43^ BIX is an index for DOM sources. A BIX < 0.7 represents important terrestrial contributions, while a BIX > 1 is characteristic of DOM with a biological/aquatic bacterial origin.^44^

The PARAFAC analysis for this study contained five components. Components C1_humic_ (Ex/Em: 235(365)/484) and C3_humic_ (Ex/Em: 230/398) had broad excitation and emission spectra, with excitation and emission maxima in the ultraviolet and visible region, respectively. They are traditionally referred to as humic-like components ^40^. Component C2_autoDOM_ (Ex/Em: 240(300)/342) had a fluorescence signal which is similar to free and protein-bound amino acids and has been ascribed to autochthonous DOM ^45^. Components C4 (Ex/Em: 230/306) and C5 (Ex/Em: 230(275)/340) were similar to tyrosine-like and tryptophan-like compounds.^45^ All optical analyses including PARAFAC were conducted using the software R (v4.0.2.) in Rstudio^46^ with the package staRdom (“spectroscopic analysis of DOM in R”).^47^ All corrected EEMs of seawater in the POS533 cruise (*n* = 299) were used for modeling.

The identified fluorescent components were compared with the published data on an open-access spectral database^48^ (OpenFluor, https://openfluor.lablicate.com). The database compares fluorescence data sets and determines Tucker congruence (set at 95%) as the similarity criteria between pairs of excitation and emission spectra. It provides a rapid way to test new PARAFAC models against those in the literature.

### 2.5 Statistical Analysis

The Pearson product-moment correlation and Spearman rank-order correlation statistical analysis tests between the oxidation rate constant (*k*′ at pH = 8 and *T* = 25 °C) and the measured biochemical variables were performed. A multiple linear regression (MLR) model was also applied to predict if the variability observed in the oxidation rate constants at a fixed temperature, pH, and oxygen saturated condition could be described by two or more organic spectral properties.

## 3 Results and Discussion

The Fe(II) oxidation kinetics process was studied in coastal seawater considering both a physicochemical and a biogeochemical framework. In the physicochemical context, experiments were carried out to study *k*′ over a range of pH and *T*. The goal was to verify if there were differences in the calculated *k*_cal_^′^ value from eq 4.^12,35^ In the biogeochemical context, variables associated with the presence of OM were analyzed. The goal was to explain the deviations observed for *k*′ in the physicochemical context. These deviations are a consequence of the interaction between Fe and OM, expressed as ligands, L
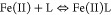
5
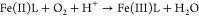
6
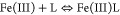
7
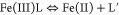
8

### 3.1 pH Effect

The dependence of log *k*′ on the pH obtained for each station was plotted (Supporting Information Figure S2) together with the expected calculated log *k*_cal_^′^ under different pH conditions. In all the experiments, linear dependences of log *k*′ with pH were observed. Deviations from log *k*_cal_^′^ as a function of pH were observed for some stations.

**Figure 2:**
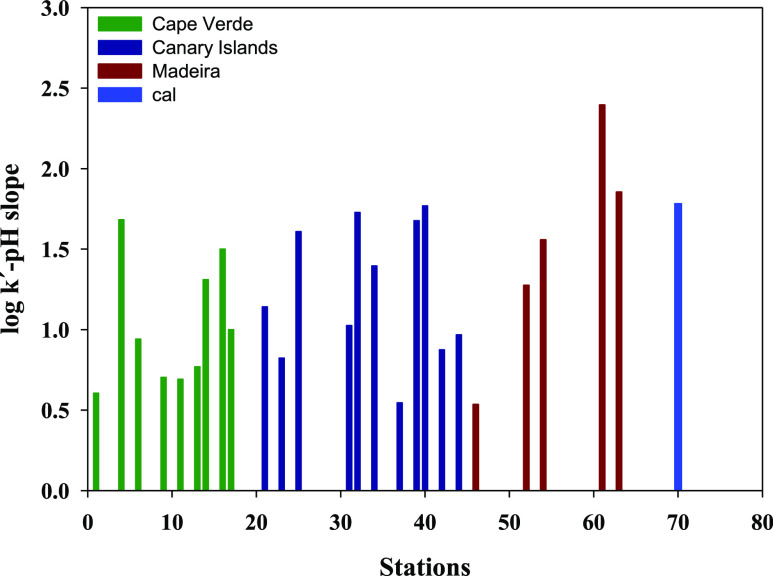
Representation of the slope of log *k*′–pH for the stations in Cape Verde, the Canary Islands, and the Madeira region. The slope was obtained from the plot of log *k*′ (min^–1^) vs pH (7.8, 7.9, 8, and 8.1) (Supporting Information Figure S2). The slope for *k*_cal_^′^ is included as St70.

The slope of the dependence of log *k*′ with the pH is shown in Figure 2. In Cape Verde, only St4 (Sao Nicolau, 1.68 ± 0.03) presented a value similar to the theoretical one (1.78 ± 0.03). The other stations presented lower slopes ranging between 0.60 ± 0.03 (St1, Santo Antao) and 1.50 ± 0.05 (St6, CVAO).

In the Canary Islands, St25 (Gomera), St32 (Tenerife-S), St39 (Gran Canaria-W), and St40 (Gran Canaria-S) presented a log *k*′–pH slope value close to the theoretical value. The other stations presented lower slopes ranging between 0.54 ± 0.03 (St37, between Tenerife and Gran Canaria) and 1.39 ± 0.08 (St34, Tenerife-E). In the north of the Canary Islands, the ESTOC site presented a slope of 0.97 ± 0.06.

Five stations were studied in the Madeira region. Station 45 (Selvagens) presented the lowest log *k*′–pH slope of 0.53 ± 0.03. At St54 (Ridge), the value was 1.56 ± 0.09, and St63 presented a slope (1.85 ± 0.03) similar to the theoretical one, and St61 presented the highest slope of the cruise (2.39 ± 0.06).

Two factors must be considered when interpreting the results. First, pH can change the speciation of both Fe(II) and organic ligands. Second, pH can affect the Fe(II)L complexation. The oxidation of Fe(II) in the absence of ligands is affected by pH as a result of speciation changes as demonstrated in previous research.^34^ In seawater, degraded and recently produced organic compounds are present and can also interact with Fe(II). The log *k*′–pH slope shows the net effect of organic ligands on *k*′. If there is an interaction between the ligands and Fe(II), forming complexes with different strengths, there will be a displacement of the log *k*′–pH slope with respect to the calculated value. The slope will shift up or down if the net result produces an acceleration or a slow-down of the oxidation kinetics rate. Moreover, if the pH changes the speciation of these complexes, a change in slope will occur. This change is due to the net effect of the organic matter matrix. However, the scientific community is currently not able to define the specific functional groups involved. In this sense, previous studies^18,49^ found differences between the effects of allochthonous and autochthonous DOM on the Fe(II) oxidation rate constant.

### 3.2 Temperature Effect

The *k*′ dependence with temperature was studied, and the activation energy (*E*_a_) was calculated using the Arrhenius equation (eq 9).
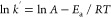
9

**Figure 3:**
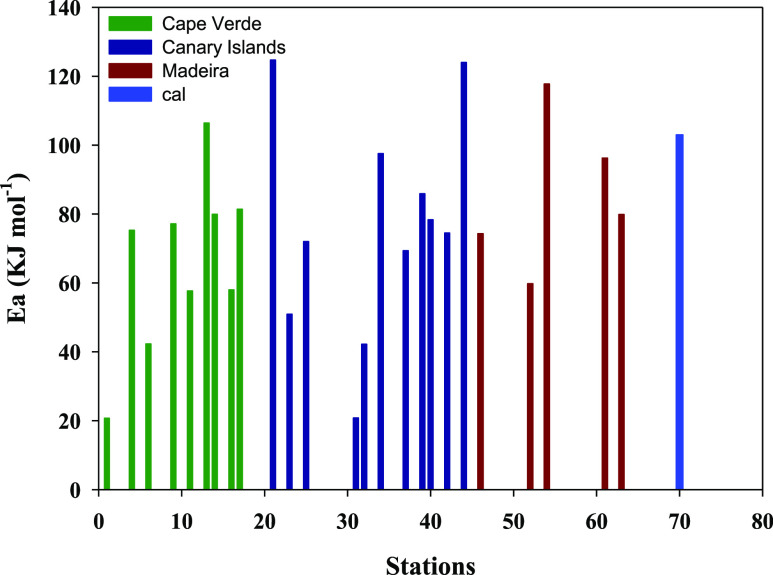
Activation energy for each station. The calculated value is also included as St70.

The ln *k*′ versus 1/*T* for ESTOC, CVOO, and Selvagens is shown in the Supporting Information (Figure S3). The *E*_a_ for each studied station was plotted in Figure 3 and compared to the calculated *E*_a,cal_ (103 ± 3 kJ mol^–1^) obtained from eq 4. For Cape Verde, the *E*_a_ ranged from 20.7 kJ mol^–1^ in St1 (Santo Antao) to 106.4 kJ mol^–1^ in St13 (Boa Vista W), the latter having an *E*_a_ within the theoretically calculated *E*_a_. In the Canary Islands, St21 (El Hierro-SE) and St44 (ESTOC) presented an *E*_a_ (124.7 and 124.0 kJ mol^–1^, respectively) higher than the theoretical *E*_a_. The other stations presented low *E*_a_ values ranging from 20.8 kJ mol^–1^ in St31 (Tenerife-W) to 97.5 kJ mol^–1^ in St34 (Tenerife-E). In the Madeira area, St54 (Ridge) presented a high *E*_a_ value (117.7 kJ mol^–1^), with coastal stations presenting 79.8 and 96.2 kJ mol^–1^ (St63 and St61, respectively).

If the rate-controlling process is always the same, then the *E*_a_ obtained from the Arrhenius equation should not change for the same experimental conditions. Different *E*_a_ may involve different mechanisms or at least, different Fe(II)-organic matter species involved in the oxidation process.

For artificial seawater, without organic ligands, or seawater in which *k*′ is not affected by the organic matter present,^35^ the average slope is −5362 ± 162 and the activation energy is 103 ± 3 kJ mol^–1^. The same results (−5434 ± 183) 104 ± 3 kJ were obtained in non-hydrothermally affected stations within the Mid-North Atlantic Ridge.^12^ According to these studies, changes in the *E*_a_ are probably caused by the interaction of organic compounds with the Fe(II) species which affected the limiting Fe(II) oxidation step. Therefore, it may have a different oxidation reaction mechanism.

### 3.3 The *t*_1/2_ at a Fixed pH and Temperature

**Figure 4:**
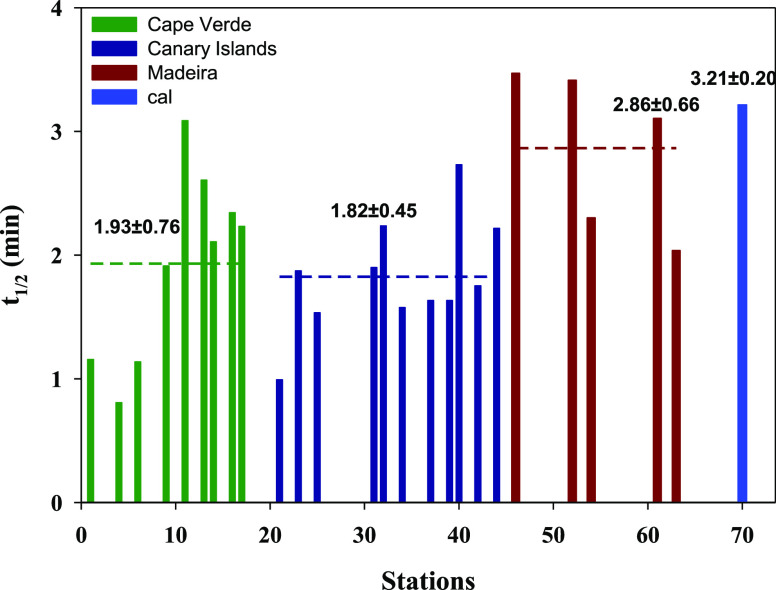
The *t*_1/2_ (min) for the stations in Cape Verde, the Canary Islands, and the Madeira region. The *t*_1/2_ theoretical value is included as St70.

Previous studies showed log *k*′ differences between stations when experiments were carried out under the same pH and *T* conditions. From the pseudo-first-order rate constant (*k*′), the *t*_1/2_ was calculated as *t*_1/2_ = ln 2/*k*′. This variable represents the persistence time of Fe(II) in each station under the studied conditions. The *t*_1/2_ was calculated for pH = 8 and *T* = 25 °C conditions (Figure 4). The mean *t*_1/2_ for the three archipelagos was 1.93 ± 0.76 min for Cape Verde, 1.82 ± 0.45 min for the Canary Islands, and 2.86 ± 0.66 min for Madeira, and the theoretical *t*_1/2_ was 3.21 ± 0.2 min. Overall, all stations presented values below the theoretical *t*_1/2_ except for St11 (3.09 min) in Cape Verde and St61 (3.10 min) in Madeira which presented values similar to the theoretical *t*_1/2_. Only two stations presented *t*_1/2_ values higher than the theoretical: St46 (3.47 min) and St52 (3.41 min).

Although factors such as O_2_, pH, and temperature are the main variables that control the oxidation kinetics of Fe(II), photo-generated compounds (organic radicals and ROS)^50,51^ and other biochemical variables can play a relevant role. When these other variables affect the oxidation process, either by accelerating or delaying it, the value obtained for *k*′ is different from the calculated *k*_cal_^′^. Photochemical processes should be important in samples from the top few centimeters of the seawater column, while thermal processes control Fe transformation in deeper waters.^50^

Variations in nutrient or DOC concentrations, the nature of the OM, and colloids present in the solution can lead to changes in the Fe(II) oxidation rate.^12,23,52,53^ These factors were analyzed for their relationship with the observed changes in *t*_1/2_ in different stations at fixed pH and *T*.

### 3.4 Nutrients

The stations in the Cape Verde archipelago presented the highest and most variable nutrient concentrations, ranging from 0.03 to 1.16 μM for total inorganic nitrogen (nitrate and nitrite), 0.07 to 0.18 μM for phosphates, and 0.04 to 0.63 μM for silicates. The Canary Islands presented the lowest total inorganic nitrogen (0.08 ± 0.11 μM) while Madeira was characterized by the lowest phosphate (0.04 ± 0.01 μM) and silicate (0.03 ± 0.04 μM) concentrations. Concentrations for each station are shown in Supporting Information (Figure S4). The effects of nutrient concentrations, in particular N and Si, in the *t*_1/2_ are generally observed when nutrient concentrations are high, for example, in phytoplankton growing media or eutrophic environments.^52,53^ The concentrations found in these areas were low and did not exert an appreciable effect on *k*′.

### 3.5 Total Dissolved Organic C and N

The DOC and TDN concentrations varied by archipelago and oceanic stations (Supporting Information Figure S5). The mean DOC and TDN concentrations were, respectively, 91.3 ± 3.5 and 6.98 ± 0.7 μM in Cape Verde, 78.2 ± 0.4 and 5.2 ± 0.1 μM in the Canary Islands, and 73.8 ± 0.2 and 5.1 ± 0.1 μM in Madeira. A slight gradient was observed which increased from south to north between the archipelagos.

### 3.6 Particulate Organic C and N

Unlike DOC and TDN, the particulate organic C (POC) and N (PN) showed significant variations between stations of the same region (Supporting Information Figure S5). The south-to-north gradient was also observed for POC and PN with the lowest concentrations measured in Madeira, followed by the Canary Islands. The highest POC and PN concentrations were measured in Cape Verde. The differences observed between archipelagos in the particulate material suggested that variations in the content and distribution of the colloidal material could also occur.^54^ However, the samples for Fe(II) were filtered through a 0.2 μm pore size filter, where the particulate material should not affect the obtained *t*_1/2_.^12^ The colloidal material which has been demonstrated to influence the oxidation process^12^ was not analyzed due to time constraints but should be taken into account in future work.

### 3.7 Bulk of DOC and the UV-Irradiation Effect

**Figure 5:**
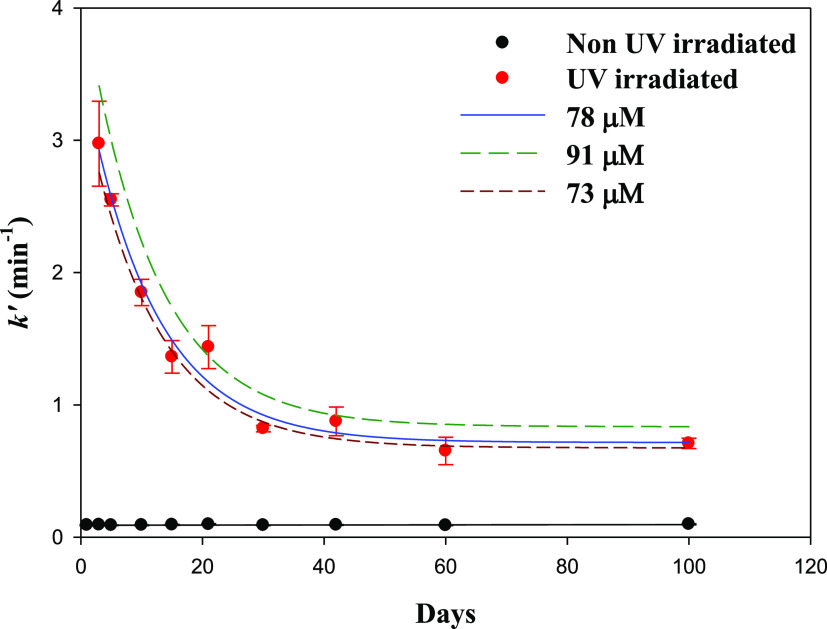
Time evolution of *k*′ for non-UV-irradiated seawater and UV-irradiated seawater for an ESTOC sample with 78 μM of DOC [(Fe(II))_0_ = 0.97 nM, pH = 8, *T* = 20 °C, *S* = 36]. The continuous line defines the behavior for a sample that contains a DOC of 78 μM as in the ESTOC. The dashed red lines represent the calculated variation that would be obtained if the DOC content changes. The longest dashed line in green corresponds to a sample that has 91 μM DOC, as in the samples from Cape Verde. The dashed line in red corresponds to a sample that has 73 μM DOC, as in the samples from Madeira.

A 20 m sea-surface water sample of ESTOC was used to carry out studies with a known initial DOC content of 78 μM. The sample was divided into two sub-samples: one was irradiated, while the other was kept in the dark (non-irradiated). The evolution of *k*′ over time was studied for 100 days at a fixed pH = 8 and *T* = 20 °C (Figure 5). For the non-irradiated seawater, *k*′ was similar during all the experiments with a value of 0.092 ± 0.003 min^–1^ and the *t*_1/2(non-IR)_ = 7.5 ± 0.2 min. For the irradiated samples, high values were obtained the first few days after the irradiation, presenting an exponential decay described by eq 10. *k*′ changed from 3.59 ± 0.10 min^–1^ on day 0 to 0.92 ± 0.02 min^–1^ on day 30 at a rate of 0.088 ± 0.01 min^–1^, after which it reached a plateau. The reduction of the initial *k*′ by 50% was reached on day 7.
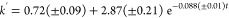
10std dev = ±0.14 min^–1^.

The study carried out with a UV-irradiated and a non-irradiated sample presented differences between the two conditions. When the organic matter undergoes photo-oxidation, ROS intermediates, ROS (H_2_O_2_, O_2_^•–^, and OH^•^) are generated and these affect the Fe(II) oxidation kinetics rate constant.^55,56^ Organic radicals such as semiquinone radicals and/or peroxyl radicals can also be generated, becoming even more important than ROS under certain conditions.^50^ Consequently, UV-irradiated samples should be left in the dark for at least 30 days to reach the plateau and measure a consistent *k*′.

The difference of *k*_(day 30)_^′^ – k(_day 0)_^′^ = 2.674 min^–1^ is a measure of the photo-generated compound effect due to the irradiation process. The effect of OM present in the sample can be calculated from the difference between the non-irradiated and irradiated *k*′_._ The *k*′ value changed by 0.82 min^–1^, which accounted for an increase in the *t*_1/2_ of 6.78 min at pH = 8 and *T* = 20 °C conditions. The effect of three DOC concentrations (73, 78, and 91 μM for Madeira, ESTOC, and Cape Verde, respectively) is represented in Figure 5 assuming a change only in the amount but not in general composition.

The competitive effect between O_2_ and H_2_O_2_ was studied in previous work,^36,57^ concluding that the oxidation of Fe(II) with H_2_O_2_ plays a relatively minor role in most natural waters. At the pH of seawater, O_2_ is the most important oxidant when [H_2_O_2_] is below 200 nM and [Fe(II)] is at nanomolar concentrations. Previous studies in the area showed that H_2_O_2_ concentrations are below 100 nM.^58^

The nature of OM should also be considered. Culture studies carried out in laboratories show a high dependence of *k*′ on the content of DOC.^59,60^ However, the same does not occur with oceanic samples^12,23^ presumably because the dilution factor in the ocean may be important.

The properties of DOM are diverse and depend on its source (terrestrial or aquatic) and diagenetic state. The colorimetric properties of DOM give information about its origin and diagenetic status.^38,44^ The analysis carried out considered the fraction of OM that absorbs ultraviolet and visible light, through the CDOM, and considered the fluorescent properties of the FDOM. The CDOM and FDOM sampling did not always coincide with the kinetic study sampling. As a result, the analysis only compares common stations or geographically close stations (Table 1).

### 3.8 CDOM and FDOM

Using spectral parameters and ratios obtained from CDOM and FDOM, different information about the characteristics of DOM was obtained (Supporting Information Figures S6–S11). The absorbances *a*_254_ and *a*_325_ are respectively proportional to the abundance of conjugated carbon double bonds^61^ and aromatic substances.^38,40^ The comparison of *a*_254_ and *a*_325_ measured for different samples did not show significant variations (Supporting Information Figure S6). For the entire study region, the *a*_254_ average value was 1.07 ± 0.23 m^–1^, with minimum and maximum values of 0.62–1.25 m^–1^. The *a*_254_ absorbances were in agreement with those obtained for temperate Atlantic waters^61^ where the higher *a*_254_ absorbances were found in surface waters (1.43 ± 0.18 m^–1^) decreasing to 0.87 ± 0.78 m^–1^ in deep waters. In this study, the *a*_325_ average was 0.11 ± 0.02 m^–1^ and ranged between 0.06 and 0.15 m^–1^. The determined coastal zone absorbances were slightly higher than those observed in oceanic waters of the North Atlantic subtropical gyre.^38^ This may be due to the increased production in coastal waters.

The *S*_R_ and E2/E3 ratios are independent of the CDOM concentration and provide information on the average characteristics (chemistry, source, and diagenesis) of CDOM.^39^ The average value for *S*_R_ (Supporting Information Figure S7) was 2.21 ± 1.29, ranging from 1.18 to 6.61, and was within the marine origin DOM *S*_R_ range. In open ocean waters, *S*_R_ varies from 1.5 to 4, while it reaches values lower than one when it has a terrestrial origin DOM.^39,62^

The E2/E3 ratio is used to track changes in the relative size of DOM molecules (Supporting Information Figure S8). As the molecular size increases, E2/E3 decreases due to stronger light absorption by high-molecular-weight CDOM at longer wavelengths.^41^ The E2/E3 ratio changed from 16 to 40 with an average of 24.39 ± 6.73. The highest ratios were obtained at St41, located close to St42 (Gran Canaria-E), while the lowest ratio was calculated at the oceanic St18.

The diagenetic state of DOM can be deduced from the BIX and HIX indexes.^44^ The BIX index varied between 0.8 and 8.1 with an average of 1.80 ± 1.81 for the studied regions. In the Canary Islands, St31 (Tenerife-W) had the highest BIX index (8.1 ± 0.75). Stations 9 (Santiago) in Cape Verde and 21 (El Hierro-SE) in the Canary Islands presented values of 0.85 ± 0.39 and 0.96 ± 0.1, respectively (Supporting Information Figure S9). However, most of the stations measured presented BIX indices greater than 1. Consequently, DOM predominantly had an autochthonous origin. Increases in the BIX index indicated a recently reworked one by bacteria DOM.^44^ The HIX index ranged between 0.09 and 0.62 with an average of 0.48 ± 0.12. In this study, HIX indexes were always below 4, indicating autochthonous DOM from a biological origin.^44^

The PARAFAC analysis characterized five components (Supporting Information Figures S10 and S11).^63^ C1 varied between 0.008 and 0.015 RU. C1 was significantly higher in the Cape Verde region (ANOVA, Tukey, *p* < 0.05). C3 ranged from 0.004 to 0.025 RU. The Cape Verde region was significantly higher than the Canary Islands.^45,64^ C2_autoDOM_ ranged from 0.004 to 0.018 RU. Two exceptions were found in the Canary Island region, at St31 and St33 (Tenerife W and E), where concentrations reached 0.54 and 0.06 RU, respectively. C4 was not significantly different between the regions (ANOVA *p* < 0.05) with an average of 0.018 ± 0.005 RU. C5 fluctuated from 0.012 to 0.12 RU with higher values at St31 (Tenerife-W) and St63 (Madeira-W) of 0.12 and 0.10 RU, respectively.

### 3.9 Statistical Analysis

The measured biochemical variables (nutrients, DOC, TDN, CDOM-*a*_254_, *a*_325_, E2/E3, *S*_R_, b–t–a–m–c peaks, FDOM-BIX, HIX, and C1–C5 from PARAFAC) at 13 stations were tested to identify any correlation with the oxidation rate constant *k*′ at pH = 8 and *T* = 25 °C. In the previous work,^12,35^ the main controlling factors in the determination of *k*_cal_^′^ were pH, temperature, salinity, and oxygen. The statistic results indicated a lack of significant correlations (*p* < 0.05) between *k*′ and the biogeochemical variables. From the MLR model, the organic variable TDN and the spectral organic variables *b*_DOM_ and C1_humic_ were able to predict *k*′ (eq 11) with an *R*-value of 0.921 and a standard error of estimate for *k*′ of 0.064 min^–1^.
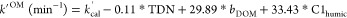
11where TDN is the total dissolved nitrogen (μM) and *b*_DOM_ is the absorbance peak that appears when protein-like or tyrosine-like components are present,^42^ and C1_humic_ is associated with humic-like components^63^ in RU (Raman units). Equation 11 was able to explain 84 ± 10% of *k*′ for the Macaronesia region (data available in Supporting Information Table S1). At fixed pH = 8, *T* = 25 °C, and *k*′ = 0.218 min^–1^, the statistical *p*-values were *p* < 0.001 for TDN, *p* = 0.015 for *b*_DOM_, and *p* = 0.009 for C1_humic_.

The equation indicated that within the highly variable DOM, compounds containing nitrogen in their structure or nitrogen functional groups could exert an important effect on *k*′. The overall effect was that increasing concentrations of tyrosine-, protein- or humic-like compounds resulted in a higher observed *k*′ than the *k*_cal_^′^. As a result, Fe(II) would have a lower *t*_1/2_ which would affect the permanence of Fe(II) in the ocean. This does not mean that only nitrogen-containing compounds have an impact on the Fe(II) oxidation process. Other functional groups have also been described in the literature.^34^ It may be that in the study area, they are the most active.

In the presence of organic ligands L with N in the structure, eq 11 can be considered and explained by eqs 5 and 6
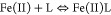

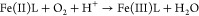


When the Fe(III)L (ferric chelate) stability constant is higher than that of Fe(II)L (ferrous chelate), oxidation of Fe(II)L to Fe(III)L by O_2_ is a highly favored reaction.^65,66^ Moreover, for ligands that have a weaker nitrogen donor, the stability of Fe(II)L increases with basicity, but not as much as that of Fe(III)L.^65^ Iron(II) adsorbed onto mineral surfaces and soluble Fe(II) chelates are important natural reductants. Studies with both Fe-goethite and Fe(II)-tiron as models of Fe(II) adsorbed and soluble Fe(II) chelates with different N–O containing compounds indicated that both the amino-functional groups and the pyridine ring are involved in complexation. Ring-N is more strongly involved than ring-O.^66^

Specific components of the DON pool in the ocean include urea, dissolved combined and free amino acids, proteins, nucleic acids, amino sugars, and humic substances.^67^ The chemical identity of most of these compounds and the mechanisms by which they are cycled are unknown.^68^ Furthermore, DON originates from both allochthonous and autochthonous sources.^67^ In this study, DON had a higher autochthonous origin (deduced from *S*_R_). Previous studies indicated that a substantial fraction of DON in the ocean has bacterial origin such as glutamine and glutamate, with the largest proportion released by planktonic marine cyanobacteria.^69,70^

Other compounds may also interact with Fe, such as bacterial siderophores and planktonic exudates such as polysaccharides and transparent exopolymers.^5^ In the same way that microorganisms follow a seasonal cycle in surface waters, the composition and concentration of autochthonous ligands will be conditioned by seasonal variability and the characteristic organism of each marine environment.

## 4 Environmental Implications

The sources and molecular identities of DOM in the ocean are not yet fully understood, and the parameterizations of organic ligands in ocean biogeochemistry models still have significant uncertainties.^7^ It is known that the presence of DOM in seawater can affect the Fe(II) oxidation rate constant.^17,34,37^ Previous laboratory studies^35,71^ show that organic compounds can accelerate, reduce, or have no effect on Fe(II) oxidation kinetics. The *k*′ variability in the presence of organic ligands suggests that the effect of DOM on Fe(II) oxidation is dependent on the molecules and their properties. This implies that specific ligands or groups of organic compounds have to be known to understand the effect that those ligands produce on Fe(II). Studies using environmental samples show that DOM from different sources presents varying effects on Fe(II) oxidation kinetics.^17,18,37^ Oxidation rates for freshwater (e.g., river water and wastewater effluent) are generally higher than those for coastal waters.^18^ The humic-type DOM (allochthonous origin) was defined as the key factor that accelerates the Fe(II) oxidation in freshwater samples. The lower oxidation rates of coastal seawater compared with those of freshwater and organic ligand-free seawater were thought to be associated with microbially derived autochthonous DOM. Few studies have considered the redox activity of compounds that makes up part of the DOM.^72,73^ DOM is capable of acting as both an electron donor and an acceptor, keeping Fe in a redox cycle.^2^ In any case, the results show the net effect of the different organic compounds that may be present in seawater.

This study remarks the important role of DOM in the Fe(II) oxidation kinetic process and the consequences of Fe(II) persistence in the marine environment. Although the physicochemical variables pH and temperature control the Fe(II) oxidation rate in a non-oxygen limited medium, the biogeochemical context is important. The *k*′ deviation from *k*_cal_^′^ was explained through the spectral characterization of the organic matter. The observed variability of *k*′ was correlated with the TDN and two spectral variables, *b*_DOM_ and C1_humic_. However, it is necessary to indicate that the ratio of the organic ligand and Fe(II) will influence the fraction of Fe(II) complexed by organics and thereby the Fe(II) oxidation kinetics. Although we have obtained a correlation and it is an important advancement, studies related to the concentration ratio between Fe (II) and organics are necessary to the extent of the validity of the empirical equation to a range of Fe(II) concentration.

The nature of DOM in the medium may control the redox cycle of Fe. In the continental margin, Fe(II) is influenced by allochthonous contributions (i.e., rivers, marshes, and estuaries). However, around the volcanic islands, DOM has an autochthonous origin. The studied archipelagos presented common characteristics: they have a volcanic origin and are generally arid. There are no fluvial contributions in these islands, but sporadic contributions through the ravines. Rainfall is quite scarce in Cape Verde and the Canary Islands. This produces a predominantly autochthonous DOM. Little is known about the compounds or structures that make up autochthonous CDOM in the ocean. This study demonstrated that a higher degree of specificity in the OM characterization is required if we want to determine the role that organic compounds play in the persistence of Fe(II) in seawater and the biogeochemical cycle of Fe..

## Supporting Information Available

[Fe(II)] and ln [Fe(II)] versus time under different pH and T conditions; dependence of log *k*′ with the pH; ln *k*′ with 1/*T*; detailed sampling protocol analysis; figures for nutrients, DOC, POC, TDN, PON, CDOM, and FDOM; and TDN (μM), b (RU), and C1 (RU) data (PDF)

## Notes

The Fe(II) database is available in the PANGAEA database as J.M.S.-C; B.Q. (2021): Fe(II) kinetic data from water samples during POSEIDON cruise POS533 (AIMAC). PANGAEA, https://doi.pangaea.de/10.1594/PANGAEA.934051. Online database OpenFluor is available at https://openfluor.lablicate.com. The PARAFAC model for this study is named “Macaronesia_POS533”.
