# Supplementary material for: Exploring the Effects of Organic Matter Characteristics on Fe(II) Oxidation Kinetics in Coastal Seawater
Source: Environ Sci Technol. 2022 Jan 25;56(4):2718–28. doi: 10.1021/acs.est.1c04512 (PMC8851691; doi:10.1021/acs.est.1c04512)
Supplement: Supplementary file 1 — es1c04512_si_001.pdf [file es1c04512_si_001.pdf]

SUPPORTING INFORMATION

Exploring the effects of organic matter characteristics on Fe(II) oxidation kinetics in coastal seawater

J. Magdalena Santana-Casiano[1*], David González-Santana[1,2], Quentin Devresse[3], Helmke Hepach[3], Carolina Santana-González[1], Birgit Quack[3], Anja Engel[3], Melchor González-Dávila[1]

[1]Instituto de Oceanografía y Cambio Global. Universidad de Las Palmas de Gran Canaria. Campus de Tafira. 35017. Las Palmas. Spain

[2]Univ Brest, CNRS, IRD, Ifremer, LEMAR, F-29280 Plouzane, France.

[3]GEOMAR – Helmholtz Centre for Ocean Research Kiel, Düsternbrooker Weg 20, 24105 Kiel, Germany

Table of contents

1. Sampling protocol and analysis

    1.1 Nutrients

    1.2 DOC, POC, TDN, PON

    1.3 CDOM and FDOM

    1.4 References

2. Supporting Figures. S1-S11

3. Supporting Table S1

Number of page (14), figures (11) and tables (1)

1. Sampling protocol and analysis

1.1 Nutrients

Samples for nutrient analysis, Nitrate+nitrite (NO_3+NO_2), phosphate (PO_4) and Silicates, usually in triplicates, were collected in 14 ml polyethylene sampling tubes and immediately frozen for later analysis (985 samples).

NO_3+NO_2, PO_4 and Silicates were analysed photometrically with a QuAAtro auto-analyser (SEAL Analytical, UK) according to previously described methods [1] and SEAL Analytical operation manual (Q-064-05 Rev 8). Precisions of the measurement were determined by averaging the standard deviation of all triplicates in that range: >50% for < 0.1 μmol L^{-1} , 20% for >0.1<5.0 μmol L^{-1}, 10% for >5.0 μmol L^{-1} for NO_3+NO_2 and 20% for< 0.3 μmol L^{-1}, 10% for >0.3 μmol L^{-1} for PO_4 and 20% for < 0.3 μmol L^{-1}, 10% for >0.3 μmol L^{-1} for PO_4 and 30% for < 1 μmol L^{-1}, 10% for >1 μmol L^{-1} for Silicate . The detection limits for the nutrient measurements were 0.03 μmol L^{-1} for NO_3, 0.02 μmol L^{-1} for NO_2, 0.03 μmol $L^{-1}$for PO_4 and 0,1 μmol L^{-1} for Silicate.

1.2 DOC, POC, TDN, PON

For dissolved organic carbon (DOC) and total dissolved nitrogen (TDN), about 20 mL of seawater were filtered through 0.45 μm GMF filters, acidified using 20 μL of 30 % ultrapure hydrochloric acid (Suprapur®), and stored in pre-combusted glass ampules at +4 °C for later analysis. About 1-3 L of seawater were filtered through pre-combusted GF/F filters (Whatman, 25 mm) for determining particulate organic carbon (POC) and particulate nitrogen (PN). Filters were stores at -20 °C for later analysis back in the laboratory.

DOC and TDN samples were analyzed by a high-temperature catalytic oxidation method applying the high-temperature catalytic oxidation method (TOC-VCSH, Shimadzu) [2].

TDN was determined simultaneously with DOC using the TNM-1 detector on the Shimadzu analyzer.

POC and PN filters were soaked in 0.1 mol L^{-1} hydrochloric acid and dried at 60 °C for removal of particulate inorganic carbon (PIC), and then analyzed using a Carlo Erba CHN elemental analyzer [2].

1.3 CDOM and FDOM

For CDOM and FDOM, 40 mL samples were collected into combusted (450°C, 8 h) amber-glass vials after filtering through 0.2 mm polyethersulfone syringe filters (CHROMAPHIL® Xtra PES-45/25, MACHEREY-NAGEL GmbH & Co.KG) and stored at 4°C in the dark until further analyses. All samples were taken in duplicate.

Samples were brought to room temperature (≈19°C) before analyses. The CDOM absorbance was measured using a Shimadzu® 1800 UV-VIS double beam spectrophotometer with a 5 cm Quartz SUPRASILVR precision cell (Hellma® Analytics). Absorption was analyzed between 230 and 750 nm in 1 nm wavelengths intervals against Milli-Q water as a reference. CDOM absorbance spectra were converted to absorption coefficients according to [3]:

$$a_{CDOM}(\lambda) = \frac{2.303A(\lambda)}{L}$$

Where $a_{CDOM}(\lambda)$ is the absorption coefficient at wavelength λ (m^{-1}), A(λ) is the absorbance value at same wavelength and L is the effective optical path length (m).

For the determination of FDOM, 3D-EEM fluorescence spectroscopy was performed using a Cary Eclipse Fluorescence Spectrophotometer (Agilent Technologies) equipped with a xenon flash lamp [4]. The fluorescence spectra for samples were measured in a 4-optical window 1 cm Quartz SUPRASIL® precision cell (Hellma® Analytics). Blank-3D fluorescence spectra and Water Raman scans were performed daily using an Ultra-Pure

Water Standard sealed cuvette (3/Q/10/WATER; Starna Scientific Ltd). The experimental wavelength range for sample and ultrapure water scans was 230 to 455 nm in 5 nm intervals on excitation and 290 to 700 nm in 2 nm intervals on emission. The Ex/Em scans were later cut to intervals from 230 to 455 nm for excitation and from 290 to 550 nm for emission in order to reduce potential noise during parallel factor analysis (PARAFAC) analysis. Samples were normalized to the area of the ultra-pure water Raman peaks [5], Rayleigh scatter was deleted from EEMs, inner filter effected were corrected and Raman scatter was removed by subtracting blanks EEMs from samples EEMs [6,7]. By using corrected EEMs, the humification index (HIX) and the biological index (BIX) were determinated (Zsolnay et al. 1999; Huguet et al. 2009). All optical analysis including PARAFAC were conducted using the software R (v4.0.2.) in Rstudio, v1.1.414 [8] with the package staRdom ("spectroscopic analysis of DOM in R") [9] . All corrected EEMs of seawater in the POS533 cruise (n = 302) were used for modeling. Five fluorescent components (SI Figure S10) were determined based on the split-half validation and compared with literature using the online database OpenFluor.

1.4 References

(1) Grasshoff, K.; Kremling, K.; Ehrhardt, M. *Methods of Seawater Analysis Wiley*; Wiley-VCH: Weinhein, 1999.

(2) Engel, A.; Galgani, L. The Organic Sea-Surface Microlayer in the Upwelling Region off the Coast of Peru and Potential Implications for Air-Sea Exchange Processes. *Biogeosciences* **2016**, *13* (4), 989–1007. https://doi.org/10.5194/bg-13-989-2016.

(3) Bricaud, A.; Morel, A.; Prieur, L. Absorption by Dissolved Organic Matter of the Sea (Yellow Substance) in the UV and Visible Domains. *Limnol. Oceanogr.* **1981**, *26* (1), 43–53. https://doi.org/10.4319/lo.1981.26.1.0043.

(4) Loginova, A. N.; Thomsen, S.; Engel, A. Chromophoric and Fluorescent Dissolved Organic Matter in and above the Oxygen Minimum Zone off Peru. *J. Geophys. Res. Ocean.* **2016**, *121* (11), 7973–7990. https://doi.org/10.1002/2016JC011906.

(5) Lawaetz, A. J.; Stedmon, C. A. Fluorescence Intensity Calibration Using the Raman Scatter Peak of Water. *Appl. Spectrosc.* **2009**, *63* (8), 936–940. https://doi.org/10.1366/000370209788964548.

(6) Murphy, K. R.; Stedmon, C. A.; Graeber, D.; Bro, R. Fluorescence Spectroscopy and Multi-Way Techniques. PARAFAC. *Analytical Methods*. The Royal Society of Chemistry December 7, 2013, pp 6557–6566. https://doi.org/10.1039/c3ay41160e.

(7) Lakowicz, J. R. Introduction to Fluorescence. In *Principles of Fluorescence Spectroscopy*; Springer US, 2006; pp 1–26. https://doi.org/10.1007/978-0-387-46312-4_1.

(8) Ihaka, R.; Gentleman, R. R: A Language for Data Analysis and Graphics. *J. Comput. Graph. Stat.* **1996**, *5* (3), 299–314. https://doi.org/10.1080/10618600.1996.10474713.

(9) Pucher, M.; Wünsch, U.; Weigelhofer, G.; Murphy, K. R.; Hein, T.; Graeber, D. StaRdom: Versatile Software for Analyzing Spectroscopic Data of Dissolved Organic Matter in R. *Water (Switzerland)* **2019**, *11* (11), 1–19. https://doi.org/10.3390/w11112366.

2. Supporting Figures

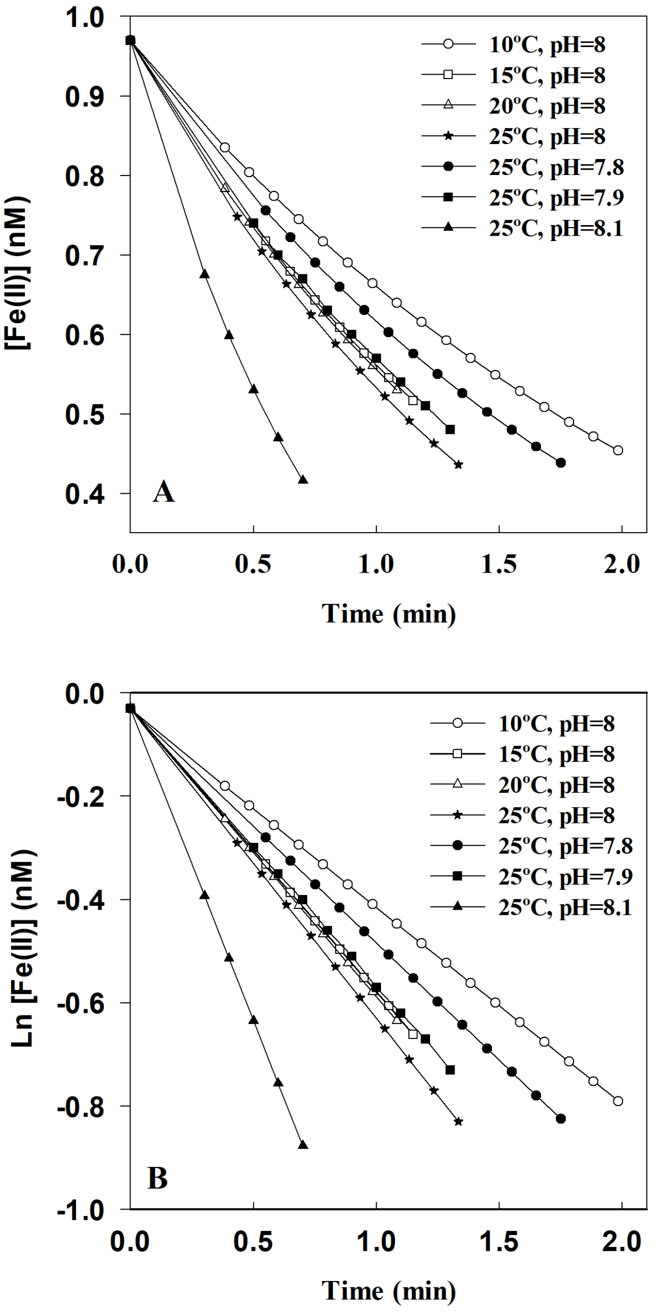

Figure S1. A) The [Fe(II)] vs time is represented at different pH and temperature conditions. B) The ln [Fe(II)] vs time is represented for different pH and temperature conditions to show that a pseudo-first order is verified.

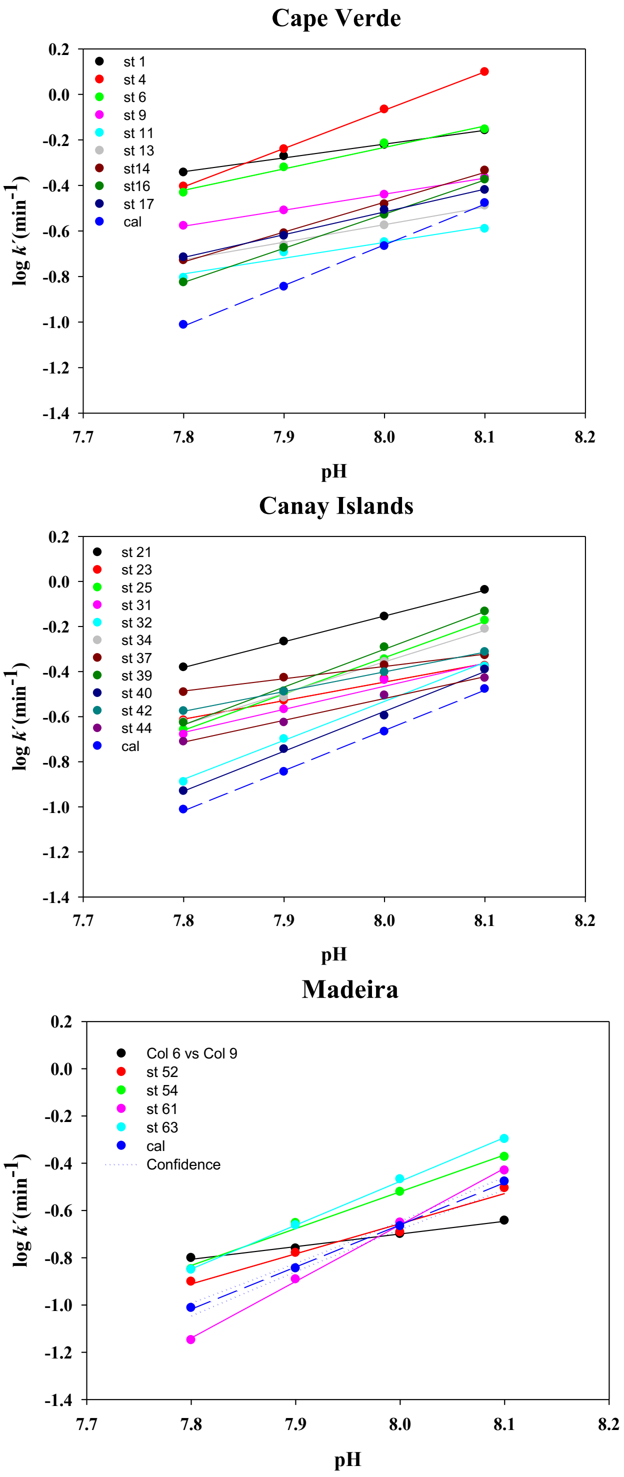

Figure S2. Dependence of log k' with the pH for the stations in A) Cape Verde, B) Canary Islands, C) Madeira region

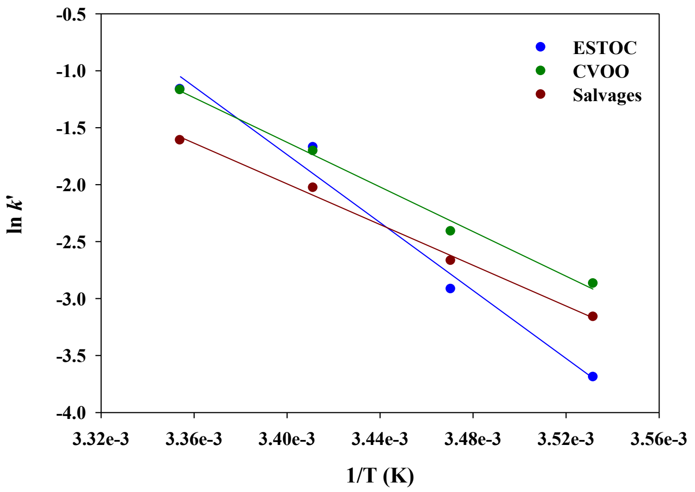

Figure S3. Arrhenius representation for the CVOO, ESTOC and Selvages stations.

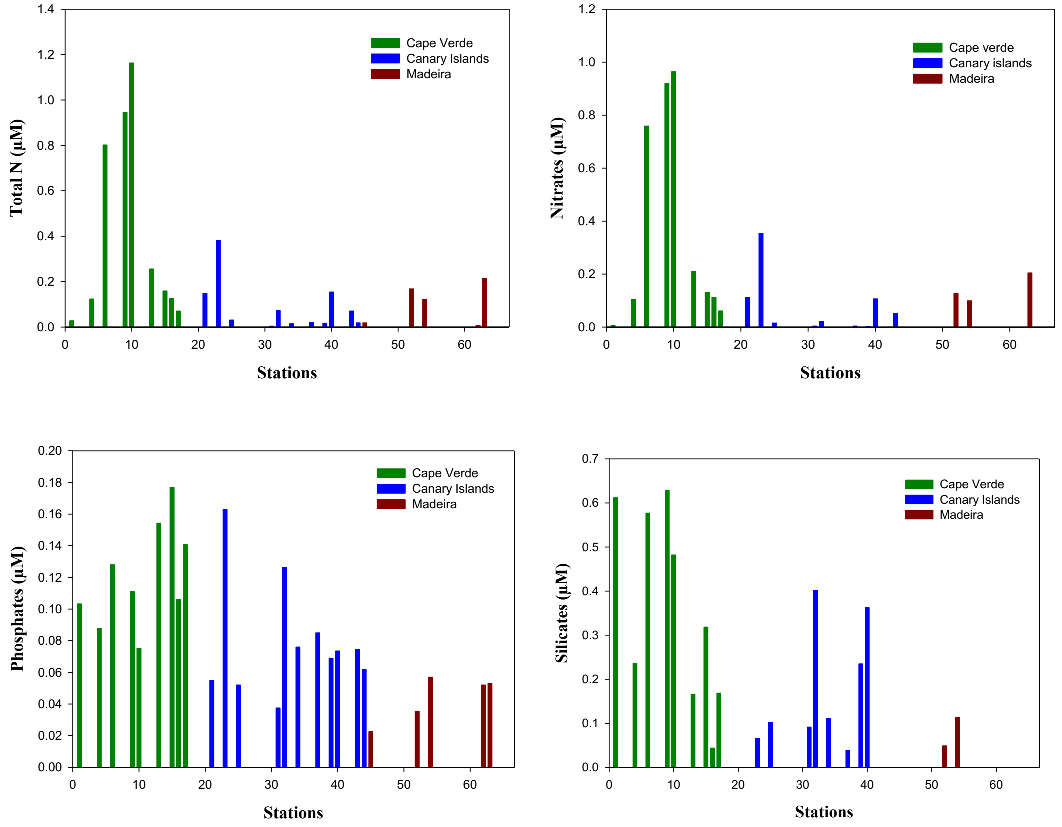

Figure S4. Total nitrogen, nitrate, phosphate and silicates in µM for stations in Cape Verde, Canary Islands and Madeira.

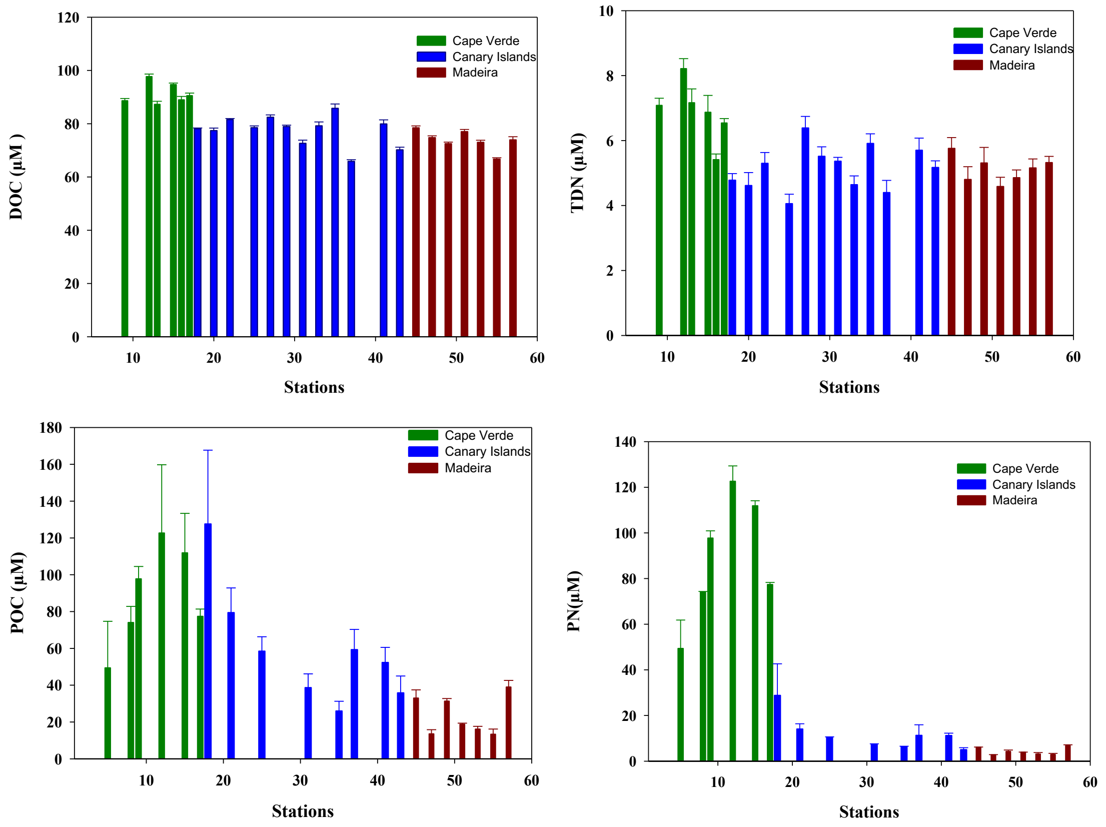

Figure S5. Dissolved organic carbon (DOC), Total dissolved organic nitrogen (TDN), particulate organic carbon (POC) and particulate organic nitrogen (TD) for stations in Cape Verde, Canary Islands and Madeira.

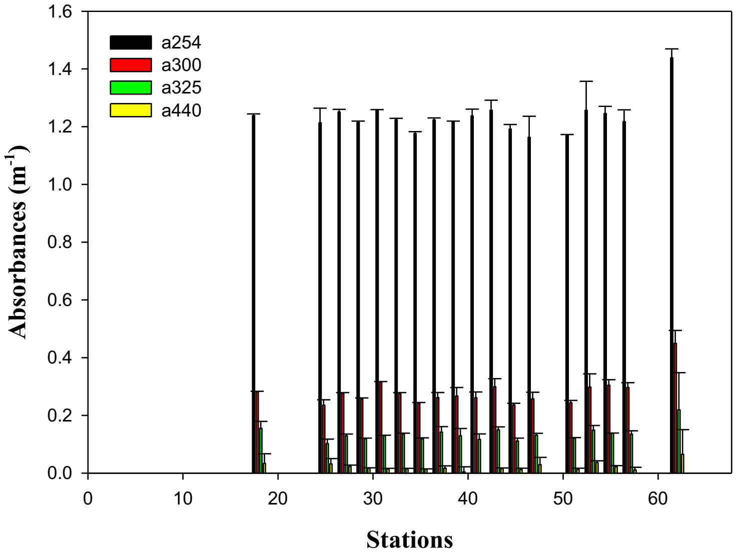

Figure S6. Absorbances a_{254}, a_{300}, a_{325}, a_{440} at λ 254, 300, 325 and 400 nm for stations in Cape Verde, Canary Islands and Madeira.

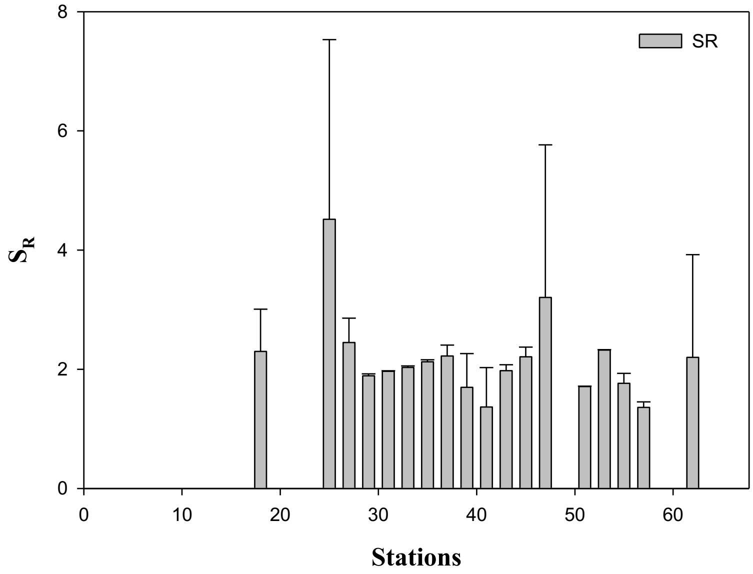

Figure S7. S_R for stations in Cape Verde, Canary Islands and Madeira.

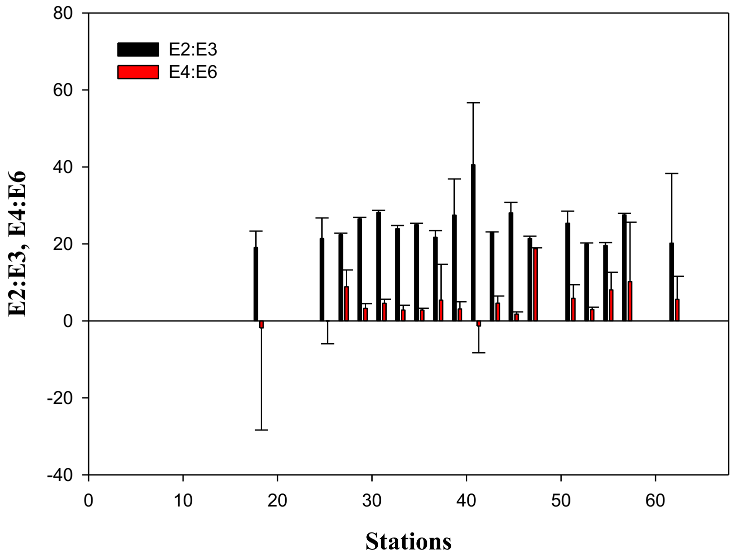

Figure S8. Absorbance ratio E2:E3 and E4:E6 for stations in Cape Verde, Canary Islands and Madeira.

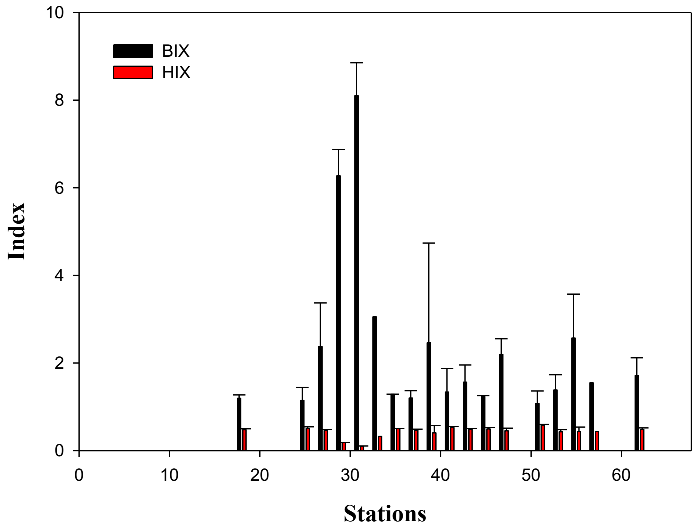

Figure S9. Bix and Hix indexes for stations in Cape Verde, Canary Islands and Madeira.

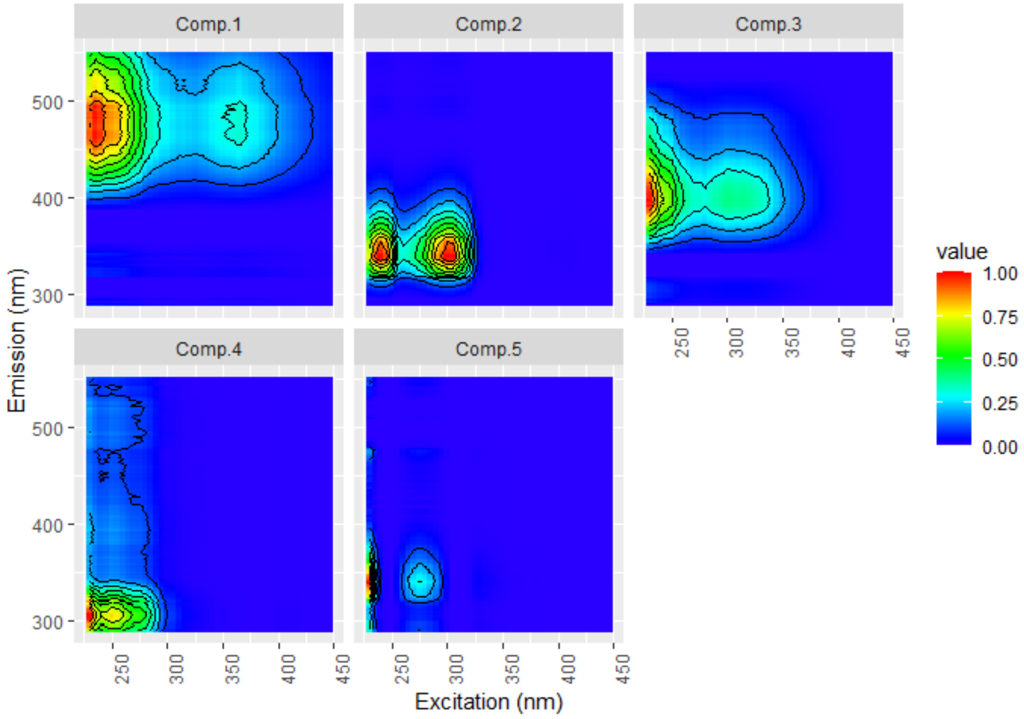

Figure S10. The spectral properties of the five fluorescent components identified by the PARAFAC analysis.

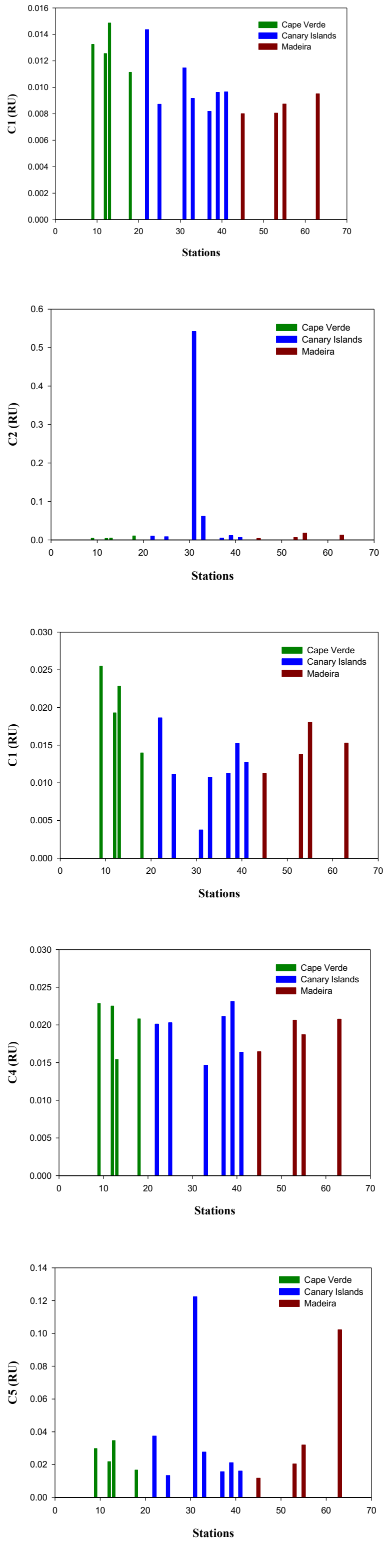

Figure S11. PARAFAC COMPONENTS, C1-C5, for stations in Cape Verde, Canary Islands and Madeira.

3. Supporting Table

Stations	k' (min^{-1})	TDN	Std Dev	b	C1	k'^{OM}_{cal}	k'^{OM}_{cal} -k'
**9**	0.3625	7.084	0.219	0.013	0.013	0.284	-0.079
**12**	0.2246	8.215	0.307	0.015	0.013	0.188	-0.037
**13**	0.2660	7.166	0.426	0.013	0.015	0.316	0.050
**17**	0.3106	6.541	0.137	0.012	0.011	0.241	-0.070
**20**	0.6989	4.615	0.399	0.015	0.014	0.652	-0.047
**25**	0.4519	4.057	0.293	0.013	0.009	0.448	-0.004
**35**	0.4401	5.913	0.294	0.017	0.009	0.396	-0.044
**37**	0.4245	4.399	0.375	0.013	0.008	0.385	-0.039
**39**	0.4245	4.830	0.3	0.012	0.010	0.372	-0.052
**43**	0.3957	5.173	0.202	0.010	0.010	0.282	-0.114
**45**	0.1998	5.760	0.333	0.010	0.008	0.142	-0.058
**53**	0.2031	4.855	0.242	0.011	0.008	0.282	0.079
**55**	0.3014	5.157	0.278	0.013	0.009	0.318	0.017
**63**	0.3405	5.250	0.3	0.012	0.010	0.312	-0.028

Table S1. Station for OM variables. TDN (µM), b (RU) and C1 component (RU) from PARAFAC used in equation (11) are included. The Std Dev for b < 0.001 and for C1 $<$ 0.006
